# Pancreatoduodenectomy for Neuroendocrine Tumors in Patients with Multiple Endocrine Neoplasia Type 1: An AFCE (Association Francophone de Chirurgie Endocrinienne) and GTE (Groupe d’étude des Tumeurs Endocrines) Study

**DOI:** 10.1007/s00268-021-06005-7

**Published:** 2021-03-01

**Authors:** Nicolas Santucci, Sébastien Gaujoux, Christine Binquet, Cynthia Reichling, Jean-Christophe Lifante, Bruno Carnaille, François Pattou, Eric Mirallié, Olivier Facy, Muriel Mathonnet, Pierre Goudet

**Affiliations:** 1 Department of Digestive and Endocrine Surgery, Dijon University Hospital, University of Burgundy, Dijon, France; 2 Department of Pancreatic and Endocrine Surgery, Cochin University Hospital, APHP, Paris, France; 3 INSERM, U1231, EPICAD Team UMR “Lipids, Nutrition, Cancer”, Dijon, France; 4 INSERM, CIC1432, Clinical Epidemiology Unit, University of Burgundy-Franche-Comté, Dijon, France; 5 Department of Hepatogastroenterology and Digestive Oncology, Dijon University Hospital, University of Burgundy, Dijon Cedex, France; 6 Department of General, Digestive and Endocrine Surgery, University Hospital of Lyon Sud and EA 7425 HESPER, Health Services and Performance Research, University Claude-Bernard Lyon 1, Lyon, France; 7 Department of General and Endocrine Surgery, Lille University Hospital, Univ. Lille, INSERM U1190, Lille, France; 8 Department of General and Endocrine Surgery, University Hospital of Nantes, Nantes, France; 9 Department of General, Digestive and Endocrine Surgery, Dupuytren University Hospital, Limoges, France; 10 Service de Chirurgie Digestive, Cancérologique et Endocrinienne, CHU « François Mitterrand », 14, rue Paul Gaffarel, 21079 Dijon Cedex, France

## Abstract

**Aim:**

To assess postoperative complications and control of hormone secretions following pancreatoduodenectomy (PD) performed on multiple endocrine neoplasia type 1 (MEN1) patients with duodenopancreatic neuroendocrine tumors (DP-NETs).

**Background:**

The use of PD to treat MEN1 remains controversial, and evaluating the right place of PD in MEN1 disease makes sense.

**Methods:**

Thirty-one MEN1 patients from the *Groupe d’étude des Tumeurs Endocrines* MEN1 cohort who underwent PD for DP-NETs between 1971 and 2013 were included. Early and late postoperative complications, secretory control and overall survival were analyzed.

**Results:**

Indication for surgery was: Zollinger–Ellison syndrome (*n* = 18; 58%), nonfunctioning tumor (*n* = 9; 29%), insulinoma (*n* = 2; 7%), VIPoma (*n* = 1; 3%) and glucagonoma (*n* = 1; 3%). Mean follow-up was 141 months (range 0–433). Pancreatic fistulas occurred in 5 patients (16.1%), distant metastases in 6 (mean onset of 43 months; range 13–110 months), postoperative diabetes mellitus in 7 (22%), and pancreatic exocrine insufficiency in 6 (19%). Five-year overall survival was 93.3% [CI 75.8–98.3] and ten-year overall survival was 89.1% [CI 69.6–96.4]. After a mean follow-up of 151 months (range 0–433), the biochemical cure rate for MEN-1 related gastrinomas was 61%.

**Conclusion:**

In MEN1 patients, pancreatoduodenectomy can be used to control hormone secretions (gastrin, glucagon, VIP) and to remove large NETs. PD was found to control gastrin secretions in about 60% of cases.

## Introduction

Multiple endocrine neoplasia type 1 (MEN1) is an autosomal dominant hereditary syndrome with a prevalence of 2/100,000 individuals. The disease is triggered by a mutation in the *MEN1* tumor suppressor gene [1–3]. The most common MEN1 lesions are (in order of frequency) primary hyperparathyroidism, neuroendocrine duodenopancreatic tumors (DP-NETs) and pituitary tumors, neuroendocrine thymic tumors, bronchic tumors, and adrenal tumors.

DP-NETs are the primary cause of MEN1-cancer-related deaths [4–6]. Surgery (pancreatoduodenectomy—PD) is recommended for large, non-functioning tumors (> 2 cm in diameter) located on the head of the pancreas because of the risk of malignant spread [7, 8]. PD may also be indicated in order to control the secretion of glucagonomas, Vipomas and insulinomas. Several authors have concluded that PD is the best option for patients with Zollinger–Ellison syndrome (ZES) because gastrinomas tend to be numerous and located in the duodenum [9–11]. Proton Pump Inhibitors (PPIs) can be an efficient, surgery-free means of controlling acid secretion, but surgery is more likely to prevent metastatic spread.

PD is a surgery with significant postoperative mortality [12]. Moreover, though MEN1-related DP-NETs tend to be slow-growing, they are often multiple and scattered throughout the pancreatic gland, meaning that there is a major risk that new tumors will develop in the portion that remains after surgery. The controversy surrounding the use of PD for MEN1 is therefore substantiated, and it seems relevant to evaluate the role of PD in MEN1 disease. The aim of this study was (1) to describe the clinical characteristics and surgical indications for PD in MEN1 patients, (2) to describe surgical complications and survival, and (3) to assess secretory control in functioning tumors with a particular focus on ZES patients.

## Methods

### Population

Our study population was extracted from the 1400-patient cohort of the *Groupe d’étude des Tumeurs Endocrines* (GTE). All MEN1 patients who underwent PD for DP-NET between 1971 and 2013 in nine hospitals with high-volume of pancreatic surgery were included in this retrospective study. Patients who underwent a previous pancreatic surgery before PD were excluded. The MEN1 cohort was approved by the Consultative Committee on Treatment of Information in Health Research (CCTIRS) and the National Committee for Data Protection (CNIL). Diagnosis of MEN1 was defined according to the clinical practice guidelines of the GTE [13]. The diagnosis of insulinoma was based on the presence of hypoglycemic symptoms associated with low plasma glucose concentrations and abnormally high serum insulin or C-peptide [14]. Zollinger–Ellison syndrome criteria were the presence of continuous specific clinical symptoms associated with ZES features found on endoscopy, an inability to discontinue high-dose proton pump inhibitors, and at least 2 out of the 4 National Institute of Health (NIH) criteria and a histological confirmation of gastrinoma [15, 16]. A diagnosis of VIPoma was confirmed in patients with the association of watery diarrhea and a high serum vasoactive intestinal peptide level. A diagnosis of glucagonoma was confirmed in patients with glucagonoma syndrome and elevated blood glucagon levels [17, 18]. DP-NETs were defined as nonfunctioning when there were no clinical symptoms of hormonal hypersecretion [8, 19].

### Recorded data

The main outcomes of the study were 90-day postoperative mortality and morbidity defined with the Dindo-Clavien classification and humoral secretion control [20]. We also analyzed the onset of metastases in the 5 years following surgery.

Postoperative mortality included all deaths occurring before hospital discharge or up to 90 days. Morbidity included any complications that appeared before hospital discharge and/or readmission within 90 days. Postoperative pancreatic fistula was defined according to the 2016 criteria of the International Study Group of Pancreatic Surgery (ISGPS) [21]. Postpancreatectomy hemorrhage and delayed gastric emptying were defined according to the 2007 criteria of ISGPS [22, 23]. Exocrine insufficiency was defined as symptoms such as steatorrhea and weight loss resolving after treatment with pancreatic enzymes. Endocrine insufficiency was defined as a fasting plasma glucose level ≥ 7.0 mmol/L and/or HbA1c > 6.5%, and/or the need to modify diet, take oral medication or insulin to control blood glucose levels.

Humoral secretion control was considered for each type of secreting DP-NET. Insulinoma, glucagonoma, and VIPoma secretions were considered to be cured if the patients had no symptoms and humoral secretions were normal. PPI use was classified into 3 categories: complete withdrawal, prophylactic treatment (e.g. prescribed after PD in order to avoid ulcerations), or maintenance of pre-surgery treatment. ZES was considered clinically cured if the patient had no recurrent symptoms without use of PPIs, probably cured when there were no recurrent symptoms but prophylactic use of PPIs, and biochemically cured when there were normal concentrations of fasting gastrin and/or the secretin stimulation test was negative for gastrin.

The following variables were collected for all patients: date of birth, gender, dates of MEN1 and DP-NET diagnosis, date of PD, DP-NET characteristics at that time, number of duodenopancreatic lesions, presence of lymph nodes, occurrence of distant metastases and the status of the JunD transcription factor.

### Statistical analyses

Continuous variables were described using means ± standard deviation (SD) or medians and ranges when appropriate. Qualitative variables were expressed in percentage. The 90-day mortality rate and complications were also expressed as percentages. Overall survival was defined as the time from surgery to death (all causes). Five-year and ten-year overall survival were estimated with the Kaplan–Meier method. All statistical tests were two-sided, and statistical significance was set at *p* < 0.05. Data were analyzed with STATA 12 statistical software.

## Results

**Fig. 1:**
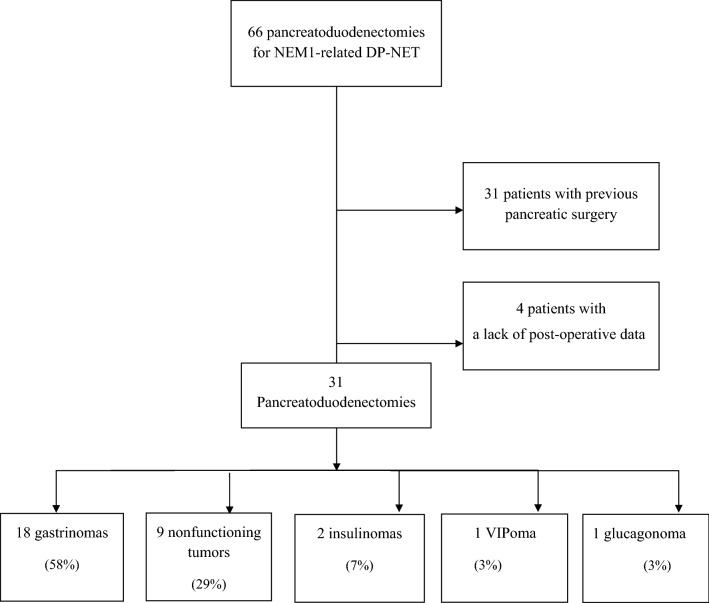
Flow chart

We analyzed 31 MEN1 patients (47% of all PDs in the GTE cohort) who underwent PD for DP-NET (Fig. 1). Trends in demographic data, associated lesions at the time of surgery, results of genetic testing and the main indications for surgery are displayed Table 1. A summary of patient characteristics, tumors, postoperative course, and follow-up are displayed in Table 2. Briefly, among the 31 patients, 18 (58%) underwent PD for ZES, 9 (29%) for a nonfunctioning PNET > 2 cm in size, 2 (7%) for insulinomas, one (3%) for a VIPoma and one (3%) for a glucagonoma. In ZES patients, 12 had surgery to control acid secretion, 5 patients with positive nodal status had surgery to prevent metastatic spread, and in 1 case surgery was done because a large NET was located in the head of the pancreas. Overall morbidity was 26%—16% of these were cases of severe clinically significant morbidity (grade III or more). Five patients (16.1%) developed pancreatic fistulas. Four patients were grade B which two required radiological guided drainage at seventh and eighth postoperative day without reoperation (grade IIIa). One patient was grade C and underwent reoperation at seventh postoperative day (grade IIIb).

**Table 1:** Demographic data, lesions at time of surgery and genetic diagnosis

	Until 2000		2001 and after		*p*
	*N* = 13	(%)	*N* = 18	(%)	
Sex ratio Men/total	7/13	54	8/18	44	0.6
Age at MEN1 diagnosis	43.5 ± 15.1		36.9 ± 17.2		0.3
Age at DP-NET diagnosis	42.9 ± 14.5		42..6 ± 14.9		0.9
Age at DP-NET surgery	43. ± 14.4		44..4 ± 14.3		0.8
DP-NET known before MEN1 diagnosis	5/13	38	3/18	17	0.2
pHPT at time of DP-NET surgery	7/13	54	13/18	72	0.4
Pituitary adenoma at time of DP-NET surgery	1/13	8	8/18	44	0.04
Adrenal at time of DP-NET surgery	2/13	15	3/18	17	1
Thymic NET at time of DP-NET surgery	0/13	0	0/18	0	1
Bronchic NET at time of DP-NET surgery	0/13	0	0/18	0	1
Previous gastric surgery	1/13	8	0/18	0	0.4
Index cases	4/13	31	1/18	5	0.1
Genetic tests performed	10/13	77	18/18	100	0.06
Positive MEN1 mutations found	8/10	80	16/18	89	0.6
Indication for ZES	7/13	54	11/18	61	0.7
Indication to control H + secretion among ZES	7/7	100	5/11	45	0.04
Indication for large non-functioning NET	3/13	23	6/18	33	0.7

**Table 2:** Type of neuroendocrine tumor and follow-up

No	Age/Sex	Year of surgery	Type of secretion	Location of the largest NET	Size of the largest NET	Post-operative complication	Late complications	Distant Metastasis Delay (months)		Follow-up (months)	Status	Cause of death
1	63/M	1971	ZES	Unknown	Unknown					54	Died	Cachexia
2	38/F	1981	ZES	Unknown	11 mm					433	Died	Unknown
3	48/F	1983	Insulinoma	Head	9 mm					180	Died	Epidermoid Carcinoma (Lung)
4	54/F	1987	ZES	Unknown	11 mm		Mellitus diabetes			374	Alive	
5	39/M	1991	VIPoma	Head	60 mm			Liver	17	78	Died	Metastasis
6	31/M	1992	ZES	Unknown	8 mm		Mellitus Diabetes			167	Died	Chronic Ethylism
7	46/M	1993	ZES	Duodenum	4 mm	Delayed gastric emptying	Exocrine Insufficiency			150	Died	Thymic Carcinoma
8	39/F	1995	NFT	Head	35 mm			Liver	0	204	Alive	
9	60/M	1996	ZES	Duodenum	8 mm		Exocrine Insufficiency			195	Alive	
10	26/M	1999	NFT	Head	60 mm		Mellitus Diabetes	Liver	26	182	Alive	
11	54/M	1999	ZES	Duodenum	15 mm	Fistula/delayed gastric emptying		Liver	36	180	Died	Metastasis
12	15/M	1999	NFT	Head	30 mm					155	Alive	
13	59/F	2000	Insulinoma	Head	12 mm					157	Alive	
14	25/M	2001	ZES	Duodenum	7 mm	Fistula/delayed gastric emptying/Haemorrhage	Mellitus Diabetes	Liver	13	198	Alive	
15	62/M	2002	ZES	Unknown	Unknown					178	Died	Operative*
16	40/M	2003	NFT	Head	90 mm					163	Alive	
17	17/M	2003	ZES	Duodenum	10 mm					70	Alive	
18	34/F	2004	ZES	Duodenum	12 mm					110	Alive	
19	48/M	2005	ZES	Duodenum	20 mm		Exocrine Insufficiency			156	Alive	
20	28/F	2006	Glucagonoma	Head	25 mm		Mellitus Diabetes			114	Alive	
21	34/F	2006	ZES	Duodenum	10 mm					146	Alive	
22	66/M	2006	NFT	Head	50 mm		Mellitus Diabetes	Liver	60	86	Alive	
23	57/F	2006	ZES	Duodenum	23 mm		Mellitus Diabetes	Liver	110	118	Alive	
24	18/F	2008	NFT	Head	14 mm	Fistula/Delayed gastric emptying				108	Alive	
25	28/F	2009	NFT	Head	25 mm		Exocrine Insufficiency			90	Alive	
26	44/F	2009	NFT	Head	> 20 mm	Fistula/Delayed gastric emptying/Haemorrhage				83	Alive	
27	47/F	2009	NFT	Head	25 mm					44	Alive	
28	35/M	2010	ZES	Duodenum	Unknown	Haemorrhage				0	Died	Operative
29	30/F	2011	ZES	Duodenum	10 mm					69	Alive	
30	64/F	2011	ZES	Unknown	< 20 mm		Exocrine Insufficiency			84	Alive	
31	40/M	2013	ZES	Duodenum	7 mm	Fistula/Delayed gastric emptying				43	Alive	

^*^After surgery for enterocutaneous fistula

Three patients (9.7%) showed a post-PD haemorrhage. Two were grade B with embolization and relaparotomy and one were grade C with death at the fourth postoperative day.

Six patients (19.4%) showed delayed gastric emptying. Seven patients (22%) developed diabetes mellitus and 5 patients (16%) developed pancreatic exocrine insufficiency.

During the mean follow-up period of 141 months (range 0–433), 6 patients developed distant liver metastasis. All distant metastases had a duodenopancreatic origin and occurred after a mean of 43 months (range 13–110) (Table 2). Among patients with metachroneous metastases, PD was indicated for 3 NETs which were > 40 mm and located in the head of the pancreas and for 3 ZES with gastrinomas of the duodenum. Half of these patients had positive nodes. One additional patient had preoperative undiagnosed synchronous liver metastases. The newly discovered metastases were resected during PD, and a 35 mm NET was removed from the head of the pancreas. Two patients underwent surgery for liver metastases, the first at 36 months and the second 96 months after PD. Seven patients (23%; 3 with ZES and with nonfunctioning tumors) developed new tumors in the remnant pancreas, but none had additional pancreatic surgery. Overall, 9 patients died. This included 3 deaths (33%) that were not directly due to MEN1 disease (alcohol, squamous cell carcinoma of the lung, sudden and unknown cause) and one case related to MEN1 but not DP-NETs (thymic carcinoma) (Table 2). Two deaths were directly related to DP-NET metastases (6%) following a loss of interaction of the JunD transcription factor. Mean age at death was 58 ± 10.1 years. Five-year overall survival was 93.3% [CI 75.8–98.3] and ten-year survival was 89.1% [CI 69.6–96.4]. During the follow-up all selected patients have not received additional pancreatic resections.

Long-term use of PPIs for ZES patients is shown in Table 3. Eleven patients out 18 (61%) no longer required PPIs for secretion control. Six patients remained on prophylactic PPIs in order to protect the gastroenteroanastomosis from ulcers, and five patients were able to stop taking PPIs completely without secretory complications or abdominal symptoms. Three ZES patients who developed liver metastasis required an antisecretory dose of PPIs, and 4 patients with no metastases were not biochemically cured. One metastatic patient who had been taking PPI for secretion control developed a perforated ulcer when the dosage was reduced. After a mean follow-up of 151 months (range 0–433), the rate of biochemical cured MEN-1 related gastrinomas was 61%.

**Table 3:** ZES patients requiring Proton Pump Inhibitors after surgery

No	Sex	Year of surgery	Positive nodes	Distant metastasis	Antisecretory PPIs * withdrawal	Daily use of PPIs*	Follow-up (months)	Persistent ZES***
1	M	1971	Yes	No	NA**	NA**	54	Yes
2	F	1981	Unknown	Unknown	Unknown	Unknown	433	Unknown
4	F	1987	No	No	Yes	Omeprazole 20 mg	374	No
6	M	1992	Unknown	No	Yes	No	167	No
7	M	1993	Yes	No	Yes	Omeprazole 20 mg	150	No
9	M	1996	Yes	No	Yes	No	195	No
11	M	1999	Yes	Yes	No	Omeprazole 80 mg	180	Yes
14	M	2001	Yes	Yes	No	Omeprazole 80 mg	198	Yes
15	M	2002	Unknown	No	Yes	No	178	No
17	M	2003	Yes	No	Yes	Omeprazole 10 mg	70	No
18	F	2004	Yes	No	Yes	No	110	No
19	M	2005	Yes	No	Yes	Rabeprazole 10 mg	156	No
21	F	2006	No	No	Yes	Omeprazole 20 mg	146	No
23	F	2006	Unknown	Yes	No	Esomeprazole 160 mg	118	Yes
28	M	2010	Yes	No	NA**	NA**	0.1	NA**
29	F	2011	Yes	No	Yes	No	69	No
30	F	2011	Yes	No	Yes	Lansoprazole 15 mg	84	No
31	M	2013	Yes	No	Unknown	Unknown	43	Unknown

*Proton Pump Inhibitor

**Not applicable

***PPIs withdrawal clinically impossible and/or gastrinemia not normalized without IPP

## Discussion

The management of patients with MEN1 remains controversial. The significant risk of surgery-related death should be considered when PD is indicated. However, though the growth of DP-NETs tends to be slow, these tumors are the primary cause of MEN1-cancer-related deaths [4, 5, 24]. One of the main aims of this study was to inform the clinical decision-making process, particularly for the care of patients with one or several MEN1-related NET(s) located in the duodenum or in the head of the pancreas. The results of this study showed that the clinical presentation of patients undergoing PD has changed over time. Surgery was indicated most often to control hormone secretions and secondly to remove large NETs. Surgery was often done to control gastrin secretion, and the complications were similar to those observed in other types of pancreatic surgery. (3) Finally, PD appears to be an efficient strategy for controlling gastrin secretion in patients with ZES and no distant metastases.

The study has several limitations. At present, this is the largest study to evaluate PD in MEN1 patients [9–11] but the number of patients remains low with difficulty to draw robust conclusions. The cohort involves a group of patients with a tumor syndrome, which generally involves complex treatment strategies and often more surgeries during follow-up. In this study patients who had already undergone pancreatic surgery were excluded in order to assess the specific role of PD. But during the life MEN1 patients who undergo PD can receive additional pancreatic resections and this might influence the reported outcome. This study covers a very long period (42 years). The clinical presentation of operated patients had changed over time (Table 1). MEN1 is now diagnosed earlier, and more associated MEN1 lesions are recognized at time of surgery. Moreover, cases registered before 1991 were retrospectively reviewed and the older pathological reports lacked some pertinent criteria such as grade, immunostaining or Ki67 index, and the duodenum was not always extensively screened for small or dispersed gastrinomas.

Indications for ZES should theoretically have disappeared with the appearance of PPIs in the 1990s, but this was not the case. The number of patients operated for ZES remained stable because indications for secretion control decreased and indications for cancer cure or prevention increased (Table 1). Indeed, indications versus abstention for non-functioning NETs date back to the 2000s [8]. In contrast, surgery has always been recommended for insulinoma (*n* = 2), glucagonoma (*n* = 1) and VIPoma (*n* = 1), as confirmed in the most recent guidelines [19].

PD theoretically carries a higher risk of fistula in MEN1 patients because of the soft consistency of the pancreatic gland and because the pancreatic duct and biliary tract are thin [24–26]. Present study found a pancreatic fistula rate of 16% and a post-operative mortality of 3% (one death from hemorrhage at the fourth postoperative day). These results are consistent with those of Eshmuminov’s meta-analysis (pancreatic fistula rate of 14.5% in 22,376 patients) [12]. Complications and failure to rescue after pancreatic surgery is correlate with hospital volume [27]. In our study all PD were performed in high volume centers with more than 20 pancreatic resections.

The occurrence of liver metastasis is a major prognostic factor for ZES patients [4, 5, 28]. In our study, 20% of ZES patients developed liver metastases after a mean follow-up of 151 (range 0–433) months, which is much higher than the 3% in Fraker et al. or 0% in Bartsch et al. after a mean follow-up of 104 months [29, 30]. Nevertheless, the probability of metastasis occurrence is likely variable due to the apparent heterogeneous nature of MEN1-related ZES. The NIH group reported the existence of aggressive (14% of cases) and a more common non-aggressive form (86% of cases) of ZES [15]. Patients with non-aggressive forms were found to have increased survival, even those who developed associated metastases. Finally, aggressive tumor growth was associated with significantly shorter survival in comparison with liver metastases without aggressive tumor growth. Five-year survival in patients with aggressive disease was 88% (95% CI 53–98), whereas 100% (95% CI 92–100) of patients with non-aggressive disease with or without metastases were alive at 5 years (*p* = 0.0012). These observations raise the question of whether PD could be used to prevent metastasis from developing in cases of non-agressive ZES. Unfortunately, there is currently no efficient way to define groups of ZES at a higher risk of death. In our study, five-year and ten-year overall survival was respectively 93.3% [CI 75.8–98.3] and 89.1% [CI 69.6–96.4] with a mean follow-up of 141 months. The negative effect of a JunD-LOI genotype on survival was confirmed in a 2013 study of the whole GTE cohort of 820 patients [31]. In the present study, death was directly related to the metastatic spread of DP-NETs in 2 cases (6%) with a JunD-LOI genotype. JunD-LOI status should therefore potentially be considered when making the decision to operate or not. As far as NFT-NETs are concerned, metastatic status is strongly correlated with the size of the pancreatic tumor, and large NETs have always been found in the pancreatic gland rather than the duodenum [32]. So, as expected, all the metastatic cases in our population harbored NFT-NETs larger than 20 mm.

The biochemical cure rate for the gastrinomas in our series was 61% with a mean follow-up of 151 months (range 0–433). Tonelli et al. and Lopez et al. reported 77% and 54% cure rates, respectively, but with shorter follow-up times [9–11]. Even if it is difficult to statistically compare these results, they all indicate that PD can effectively control ZES-related gastrin secretion in patients with no metastatic disease. On the other hand, stopping PPI treatment may be dangerous, particularly for metastatic patients.

This study on a relatively large cohort of MEN1 patients confirms that PD results in a rate of complications that is typical for pancreatic surgery. PD can be used to control hormone secretions (gastrin, glucagon, VIP), to remove large NETs located on the head of the pancreas and for ZES when there are associated NETs in the pancreatic head or if pathological nodes develop around the duodenum.
